# Origami paper analytical assay based on metal complex sensor for rapid determination of blood cyanide concentration in fire survivors

**DOI:** 10.1038/s41598-021-83186-0

**Published:** 2021-02-10

**Authors:** Azarmidokht Sheini, Marzieh Dadkhah Aseman, Mohammad Mahdi Bordbar

**Affiliations:** 1 Department of Mechanical Engineering, Shohadaye Hoveizeh University of Technology, 78986 Susangerd, Iran; 2 Department of Chemistry, Faculty of Sciences, Tarbiat Modares University, 4838 Tehran, Iran; 3 Independent Researcher, Personal Laboratory, 74614 Fasa, Iran

**Keywords:** Analytical chemistry, Biochemistry, Environmental chemistry, Chemistry

## Abstract

Cyanide-based blood poisoning can seriously damage fire victims and cause death if not detected quickly. Previous conventional methods require laboratory equipment, which are expensive and increase the duration of the analysis. Here, a simple origami based microfluidic device was introduced for point of need detection of blood cyanide concentration in people involved in fire. The device is made of four layers of paper. Each layer was in the size of 1 × 1 cm folded on each other. In this work, the blood sample was acidified by trichloroacetic acid to separate cyanide from methaemoglobin in the form of HCN gas. The produced gas released into borate buffer to recover free cyanide ions which interacted with the Pt complex ([Pt(*p*-MeC_6_H_4_)_2_(phen)]) used as a receptor in this study. Optimized conditions were applied to have a suitable interaction causing the color of the receptor to change from yellow to colorless. The color changes were recorded by a smartphone, and the sensor response was calculated by the routine image analysis software. The assay was capable of determining cyanide ions at different concentrations in the range of 1.0 to 100.0 µmol L^−1^. The detection limit of these determination was equal to 0.4 µmol L^−1^. The assay responses were not affected by the interfering species. As a practical analysis, the proposed sensor was applied to determine cyanide ions in the blood sample of 20 studied fire survivors and 10 controls with high accuracy.

## Introduction

Cyanide is one of the toxic substances in the fire smoke^1^. Type of materials, burning temperature and period of fire can influence the amount of cyanide^2^. Humans can be exposed to cyanide contamination through inhalation or skin adsorption of smoke^3^. The inhaled cyanide has high affinity to bind with groups of some proteins and enzymes in tissues and blood such as cytochrome oxidase and methaemoglobin causing hypoxia, respiratory disorders and even death^3^.

In fire occurrence, determination of blood cyanide concentration (BCC) is one of the goals of forensic and medical centers^4–6^. In general, BCC has been analyzed by some popular methods based on electrochemistry and chromatography^7,8^. The techniques represent reliable results, but their experiment procedures require sophisticated instruments, harmful reagents, time-consuming preparations, and skilled experts.

The colorimetric sensors were used as an appropriate alternative for rapid determination of cyanide^9–12^. Among various sensing elements employed in these sensors, metal complexes have been much considered due to having a comfortable preparation method and providing sensitive and selective responses with a simple detection mechanism^13,14^. According to this approach, detection of cyanide was performed using Zn (II) complexes^14,15^, Co (II) complexes^16^ and Cu (II) complexes^17–20^.

Recently, square-planar platinum (II) complexes have attracted considerable attention as effective chemical sensors owing to their interesting structures and other colorimetric and luminescence properties^21–23^. They have a high tendency to participate in metal–metal and π-π interactions^22,23^. Therefore, these complexes exhibit high performance in detecting different types of molecules and ions in vapor or liquid phases such as oxygen, pH, carbon dioxide, ammonia, specific metal ions and anions, toxic molecules and biomolecules^24^. In the case of cyanide, Pt exhibits high affinity to this anion and forms a stable Pt-CN complex^25^. The logarithm of the stability constant for this complex is equal to 40, being higher than that coordinated by copper, nickel, zinc or cobalt^25^. Therefore, it appears that sensor-based Pt complexes have high potential to determine cyanide ions at low concentrations in blood samples and without any interference.

The above sensing applications have some limitations in measurements such as using a difficult sample preparation process prior to BCC determination, needing large amounts of reagents and employing glassware and a complex readout device to monitor the color changes. In addition, it is impossible to use them for in situ analyses^26^.

Paper-based sensors are well-known as feasible tools in analytical chemistry owing to their attractive advantages such as simplicity in preparation, easy use, inexpensive application, compatibility with nature, portability for commercial and point of care analyses^27,28^. Owing to their flexibility, these devices can be designed in origami format^29–32^. The origami based sensor is provides a high dimensional flow that makes the sensor includes several analytical operations, such as separation, purification, and pre-concentration before sensing the sample on a single platform^29^. Therefore, the time and volume of reagents required for analysis are significantly reduced. Paper analytical device (PAD) can be combined with different transducers to implement colorimetric, fluorimetric and electrochemical applications^29,33,34^.

Cyanide in blood binds with hemoglobin^1^. By acidification of blood samples, the protein is degraded and separated from cyanide. Under acidic conditions, the released ions are protonated, causing the formation of gaseous hydrogen cyanide (HCN). The gas produced is entered to strong alkaline solution and deprotonated, so that cyanide ions are created^35^. In most of previous studies, Conway cells were used for both separation and diffusion procedures^1^. Although it is an efficient method, it still prolongs the analysis time or uses a large amount of strong acid and base, which is not safe and economical.

In summarize, there are many methods in the literature for the colorimetric determination of cyanide in the blood samples but they are not user-friendly, cost-effective and safe. These method expend a plenty of time for preparation of blood sample, need large volumes of reagents, use fragile glassware, complex instruments and strong acids and bases. These limitations are diminished in the present study by developing a new three-dimensional µPAD based on colorimetric determination of BCC. Unlike the other microfluidic structures used for detection of cyanide, the proposed µPAD is designed on a multilayer paper, which is capable of carrying out the protein degradation, hydrogen cyanide production, and cyanide ion release and identification without using Conway cells. Compared to previous colorimetric techniques, this study used a derivative of platinum (II) complexes, ([Pt(*p*-MeC_6_H_4_)_2_(phen)]), which is called as receptor. It has already proven that these complexes have high affinity to cyanide ions, therefore, it is expected that the new sensor has high sensitivity to the low concentrations of cyanide in the blood samples. In this method, we want to use weaker acids and bases to provide a safe assay. The µPAD is fabricated in the size of (1 cm × 1 cm), which can be portable. It seems that this sensor can consume less volume of analyte and reagents. However, the assay should be calibrated under optimal conditions, and its selectivity should be investigated in the presence of other species, so that it can be used to measure cyanide in the blood sample of fire survivors.

## Results and discussions

### Characterization of fabricated µPAD

The process of pattern designing and fabrication was evaluated by field emission scanning electron microscopy. The SEM images shown in Fig. S2 demonstrate that the bare paper is constructed by interwoven cellulose fibers (Fig. S2b-1). After printing and baking the paper, the ink penetrated into the paper texture and blocked the pores and masked the fibers (Fig. S2b-2). Therefore, the barrier section was resistant to water infiltration. Figure S2c indicates that the Pt complex was homogenously distributed on the surface of paper.

To confirm that the preparation of the detection zone was repeatable, five individual µPADs were filled by 1.0 µL of the receptor. The color intensities for each detection zone were determined and collected in Table S1. The relative standard error (RSD) for these measurements was calculated. The low error values demonstrate that the injection of the receptor was performed in a repeatable process.

### Sensing process

**Figure 1:**
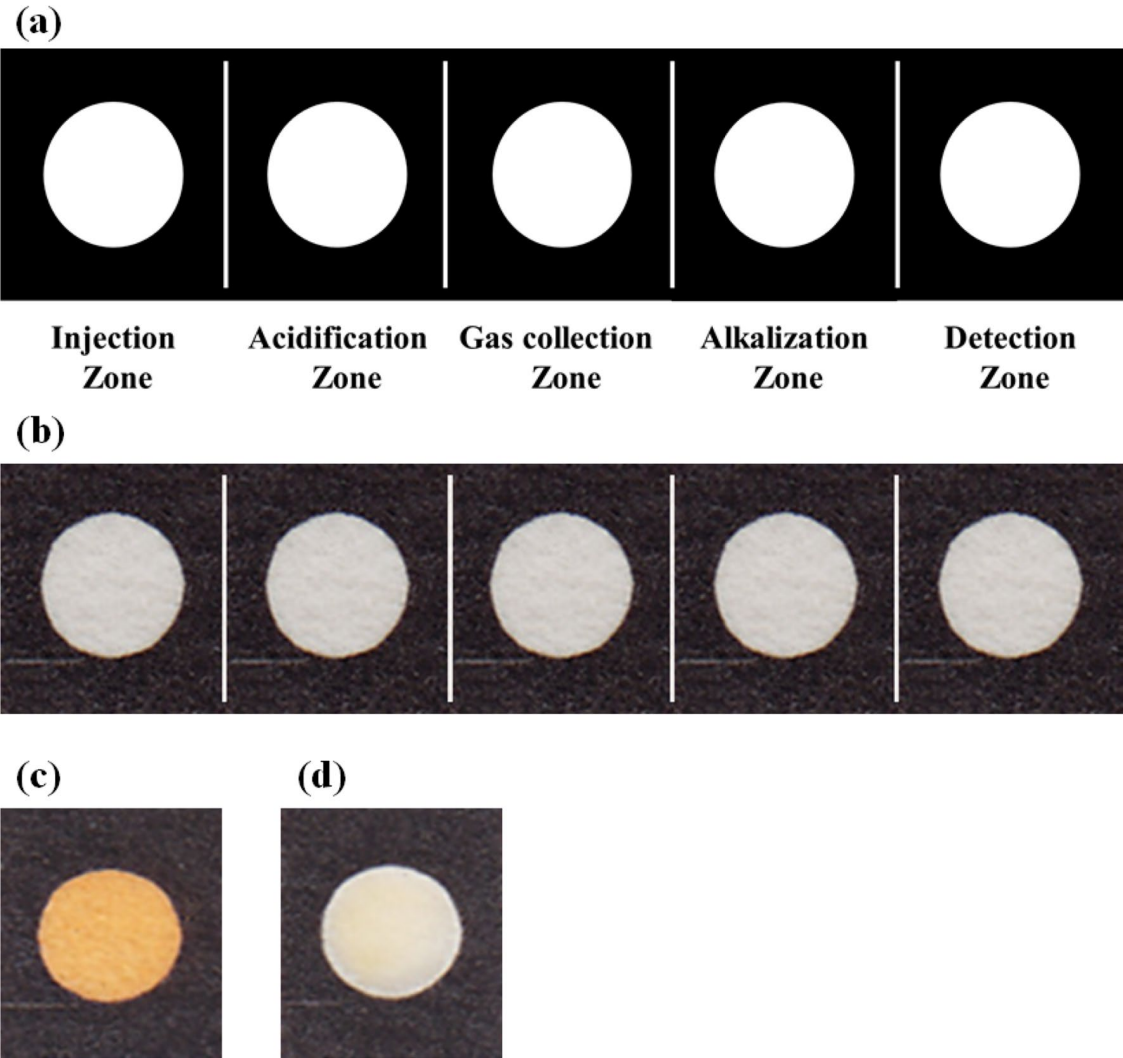
(**a**) The schematic pattern and (**b**) the image of fabricated µPAD, (**c**) the image of detection zone after injecting receptor, (**d**) the image of detection zone after exposing to 100.0 µmol L^−1^ of cyanide ions.

The injecting sample was entered to µPAD and directed to the acidification layer. In this layer, the cyanide was separated from protein and converted to protonated form HCN. This event occurred by decomposition of protein. The produced gas was followed to gas collection layer and then transferred to alkalization zone. In the alkaline media, gas was deprotonated and the cyanide ions were provided, which were moved to the detection zone and interacted with the Pt complex. Due to interaction, the color of the receptor changed from yellow color to being colorless. Figure 1c and Fig. 1d present the images of the detection zone before and after interaction. This interaction was also investigated by a UV–Vis spectrophotometer. As Fig. S2d shows, the receptor has a maximum peak at 420 nm, which decreases after interaction with cyanide ions, confirming the results of visual detection. On the basis of published reports, it seems that the cyanide ions could replace labile ligands such as Cl^−^, DMSO and SMe _2_ on Pt(II) center but the p-MeC 6 H 4 or 1,10- phenanthroline ligands in structure of complex A were not replaced with such anion. Probably, the reaction of complex A with cyanide led to formation of the Pt (IV) complex [Pt(p-MeC 6 H 4) 2 (phen)(CN) 2], (Compound B), by oxidative addition of two cyanide anions onto the Pt(II) center. The chemical reaction equation for cyanide detection is shown in Fig. S1b.

### Optimization

Different experimental parameters such as concentration of trichloroacetic acid (TCA), type, pH and concentration of buffer, amount of the receptor, number of layers for gas collection and volume of analyte can influence the mechanism of the complex formation between the receptor and the cyanide ion. These parameters should be optimized to have a stable complex and a convenient response for the proposed assay. To start the optimization, the experiment was performed by 1.0 µL of TCA (15.0% W/V), 1.0 µL of Tris buffer (0.1 M) and 1.0 µL of the receptor (1.0 mg mL^−1^).

**Figure 2:**
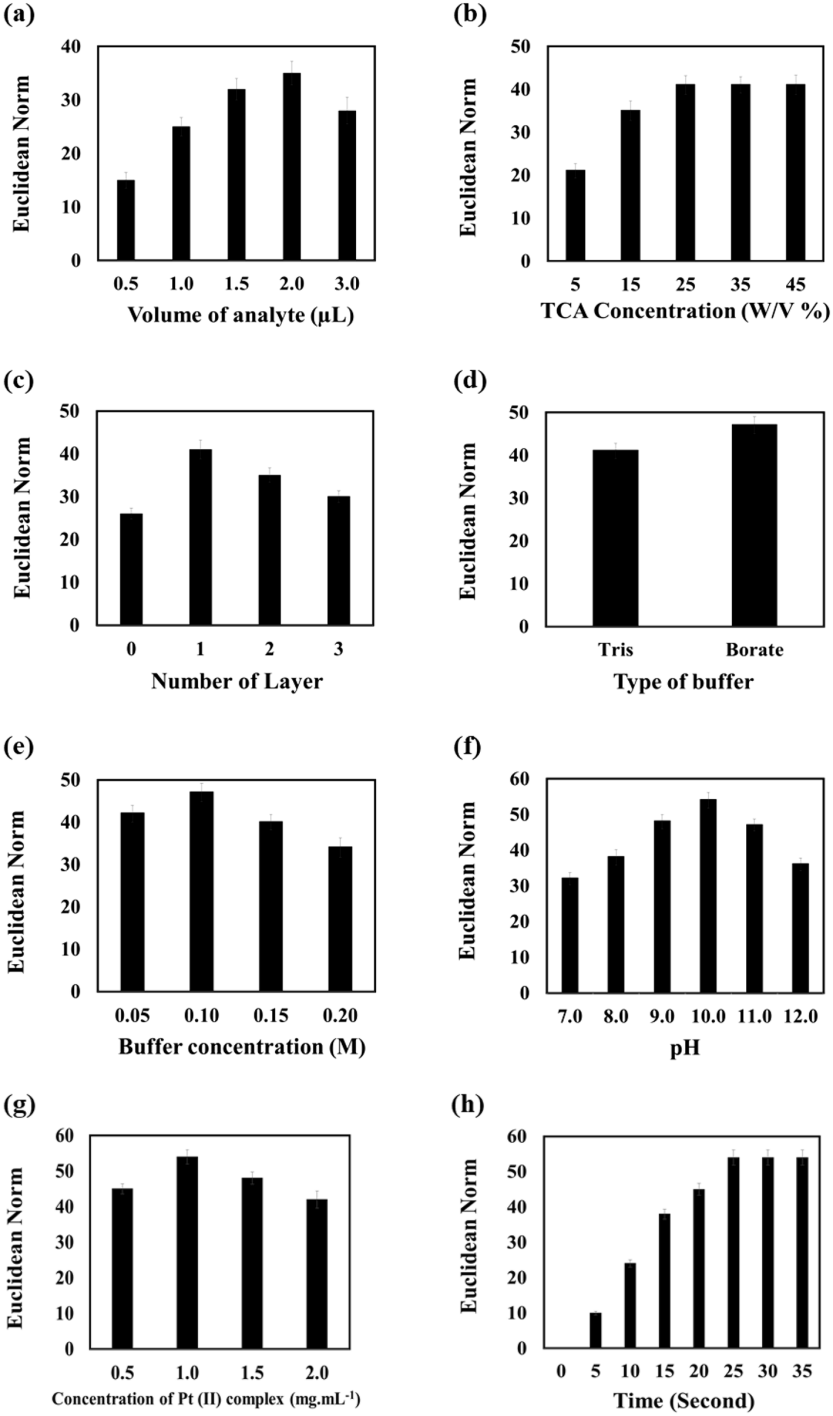
The effect of parameters including (**a**) volume of analyte, (**b**) TCA concentration, (**c**) number of gas collection layer, (**d**) type of buffer, (**e**) concentration of buffer, (**f**) pH of alkalization zone, (**g**) concentration of Pt (II) complex as receptor and (**h**) time of reaction on the response of sensor. The results were achieved in the presence of cyanide ions with the concentration of 40.0 µmol L^−1^.

In the first step, the effect of volume of analyte on the sensor response was investigated. As Fig. 2a shows, the best interaction occurred when 2.0 µL of analyte was consumed. In other words, lower volumes are not enough for passing the sample to the detection zone, and the higher volumes washed the receptors to the side of detection zone and caused a large portion of the response to be lost. For further studies, 2.0 µL of the sample was used.

TCA was employed to acidify the sample. The ability of this material for breaking the covalence bond between cyanide and protein depends on the concentration. Therefore, the mechanism of acidification was applied at different concentrations of TCA in the range of 5–45% W/V. As Fig. 2b shows, the best dissociation was observed at the concentration of 25% W/V. At higher values, no significant changes were observed in the assay responses. Therefore, 25% W/V was selected as the optimum concentration of TCA for further studies.

The gas produced in the previous step can be transferred to the next layer, immediately or during a specified interval. As Fig. 2c clearly shows, it is better to place a layer for gas collection between acidification and alkalization steps. Unlike the immediate transfer, this work helped the gas to have enough time for entering to the alkaline media, deprotonating and transforming to cyanide ions. Increase of the number of layers has no desirable effect on the response, since a portion of gas remained in the paper texture or its flow to the next step was slow, prolonging the time of analysis. Therefore, the device was fabricated by five layers.

To investigate the effect of type of buffer on the interaction of the cyanide and receptor, the examination was performed in both Tris and borate buffer (pH 11.0). As explained in Fig. 2d, borate buffer is an appropriate media for this interaction. Additionally, the concentration of buffer was evaluated in Fig. 2e. Obviously, the sensor response increases by increasing the buffer concentration up to 0.1 M. The higher concentration was not useful due to interfering of the ion-ion interaction^36^. To find the effective pH value for this interaction, the pH of buffer changed in the range of 7.0–12.0. The results were collected in Fig. 2f. As seen, the maximum response was obtained at pH 10.0. At lower pH, cyanide existed in the protonated form (HCN), so that the response of the assay was decreased. The unsuitable result at higher pH is due to hydroxyl ions interferences^36^.

The receptor (Pt (II) complex) concentration can affect the performance of the assay. For this purpose, different amounts of the receptor in the range of (0.5–2.0 mg mL^−1^) were prepared to participate in interaction with cyanide ions. As Fig. 2g illustrates, 1.0 mg mL^−1^ was a proper concentration. The complete interaction did not occur between cyanide and receptor at lower concentrations. Additionally, the intense color of the receptor at higher concentrations masked the partial differences caused by the interaction of the receptor and cyanide. Furthermore, the receptor with the concentration of 1.0 mg mL^−1^ was used.

To find the optimum time, the incubation of the receptor and cyanide was monitored 0 to 40 s. As Fig. 2h shows, the appropriate time for flowing the sample through the layers of paper and having a complete interaction with receptors was 25 s. After that, the assay responses remained constant, and no color changes occurred. Therefore, the photos of the detection zone were collected after 25 s.

### Quantitative analysis

**Figure 3:**
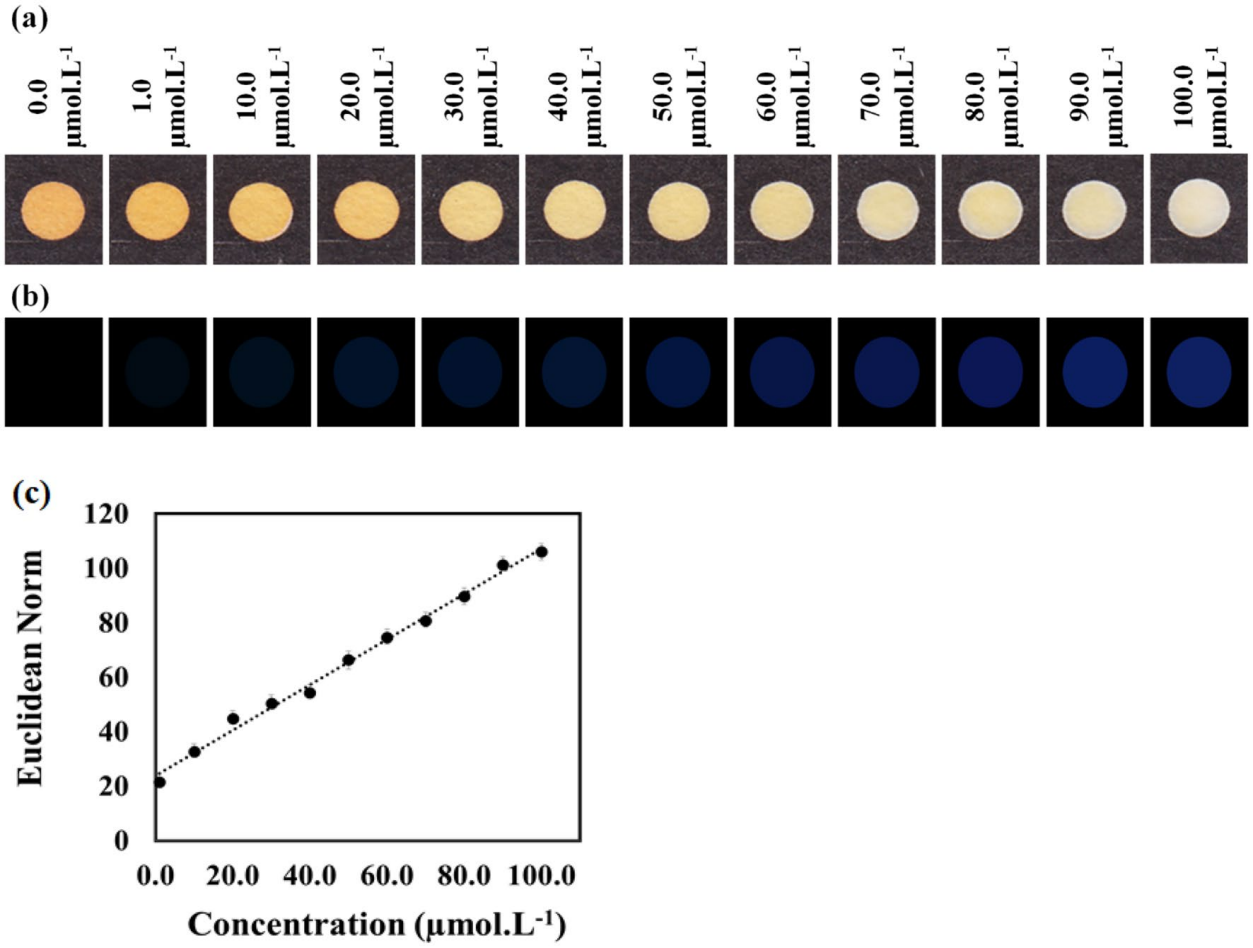
(**a**) The colorimetric responses, (**b**) the color difference maps and (**c**) the calibration curve for different concentration of cyanide ions. The study was performed in the presence of TCA (25.0% W/V), borate buffer (0.1 M, pH 10.0) and receptor (1.0 mg mL^−1^). 2.0 µL of analyte was injected and the image of sensor was captured after 25 s.

The fabricated µPAD was applied to examine the different concentrations of cyanide in aqueous solutions. The amount of cyanide varied from 0 to 100.0 µmol L^−1^. Figure 3a shows the colorimetric responses. The yellow color of the receptor gradually faded by increasing the cyanide concentration. Figure 3b depicts the changes in the color of the receptor before and after exposure to analyte as colorimetric difference maps. As seen, the color intensity was amplified when the amount of cyanide was boosted to higher values. The pure responses for each concentration were calculated and used to plot the calibration curve. As Fig. 3c shows, the relationship between the sensor responses and cyanide concentrations was linear in the range of 1.0–100.0 µmol L^−1^. The limit of detection was obtained as 0.4 µmol L^−1^. Table 1 presents the list of the analytical performance of the proposed µPAD. The results reveal that µPAD was capable of determining cyanide ions with acceptable sensitivity and a wide dynamic range.

**Table 1:** The analytical features L for determination of cyanide by using the proposed assay.

Figures of merit	Value
Linear range (µmol L^−1^)	1.0–100.0
Limit of detection (µmol L^−1^)	0.4
Correlation coefficient	0.992
Calibration sensitivity	0.8
Analytical sensitivity (for 90.0 µmol L^−1^)	0.3

### Evaluation of reproducibility

**Figure 4:**
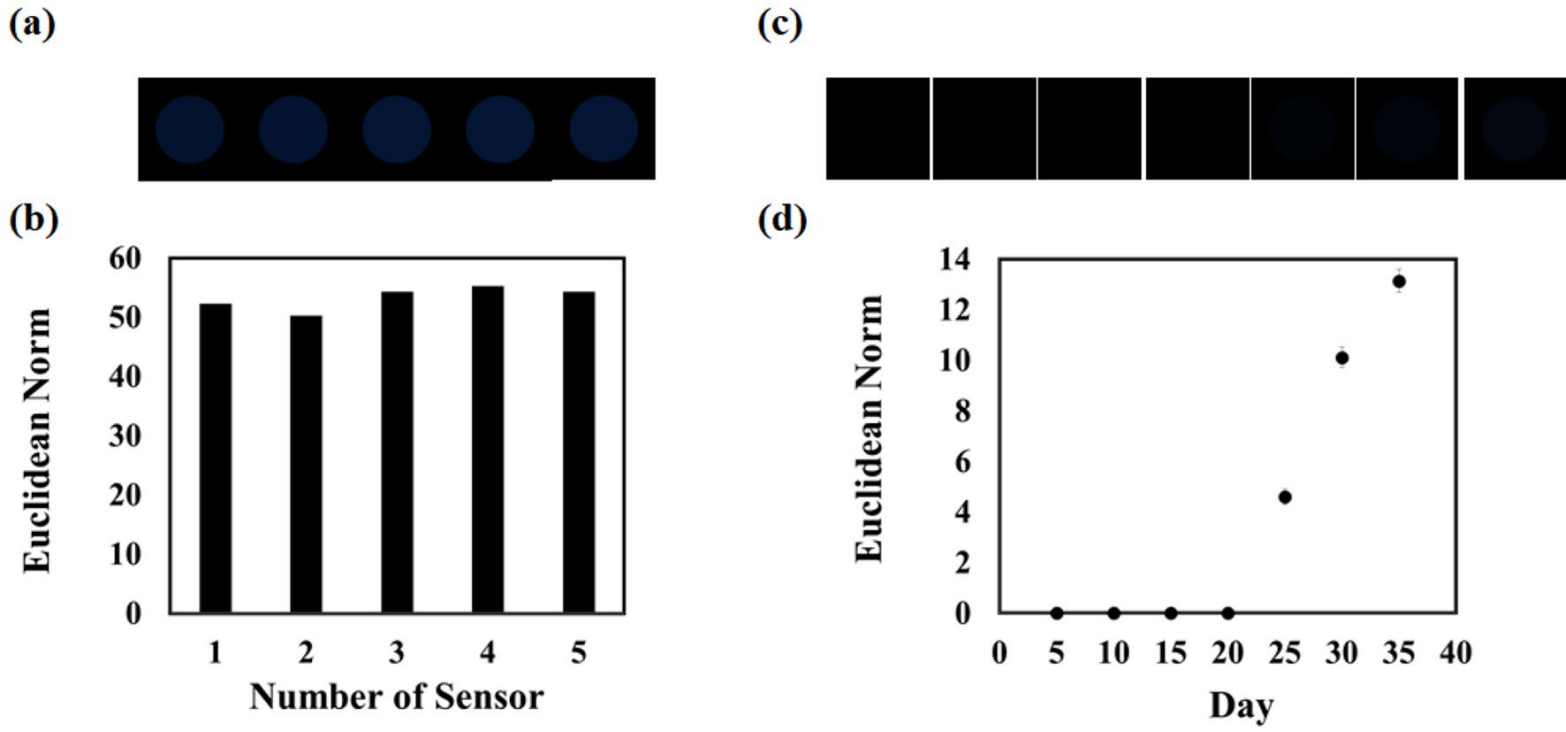
Evaluation of reproducibility of responses: (**a**) colorimetric difference maps and (**b**) numerical plot. Evaluation of the stability of fabricated µPAD: (**c**) colorimetric difference maps and (**d**) numerical plot. The study was performed in the presence of 2.0 µL of analyte (40.0 µmol L^−1^), TCA (25.0% W/V), borate buffer (0.1 M, pH 10.0) and receptor (1.0 mg mL^−1^) and the image of sensor was captured after 25 s.

Five fabricated µPADs were separately exposed to 40.0 µmol L^−1^ of cyanide ions to investigate the reproducibility of assay responses. For each µPAD, the color intensities and the Euclidean norm of response vector were determined. As Fig. 4a,b respectively illustrates the colorimetric difference maps and the bar plot which are represented the same responses for five determinations. The relative standard error was equal to 3.77%, being lower than the tolerance value (5.0%). The result indicates that the proposed assay provide reproducible responses to cyanide ions with the identical concentration.

### Stability of sensor

It is possible that the receptor will be degraded under different environmental conditions. Therefore, it is necessary to check how long the sensor is stable. For this purpose, the color intensity of the receptor was investigated for a period of time. As Fig. 4c,d shows, there were no changes in the color of the receptor for 20 days. After that, the receptor lost its durability against thermal and physicochemical variations. The cyanide ions were subjected to µPAD immediately and 20 days after the fabrication process. The results were compared to each other by statistical methods. As Table S2 illustrates, no significant difference was observed between the obtained responses. Therefore, µPAD can be stored at ambient air for 20 days before being used for analysis.

### Study of interferences

To estimate the selectivity of assay responses, µPAD was employed to detect cyanide ions (40.0 µmol mL^−1^) in the presence of different interfering materials. The amount of foreign species was 100, 50 and 20 times higher than the cyanide concentration. For this study, the effects of a number of cations (Na^+^, K^+^, Ca^2+^, Mg^2+^, Fe^3+^, Fe^2+^, Al^3+^), anions (NO_3_^−^, PO_4_^3−^, SO_4_^2−^, CO_3_^2−^, SO_3_^2−^, Br^−^, Cl^−^, I^−^ , SCN^−^) and chemical and biological compounds (uric acid, urea, nicotine, ethanol, cholesterol, cysteine, glucose, urea) were evaluated. As Table 2 clearly shows, the assay was resistant to interfering species with the concentration at least 20 times more than cyanide concentrations. It shows that a robust complex was formed between the receptor and cyanide ions, thereby enhancing the selectivity of the proposed method for the studied analyte. On the other hands, the Euclidean norm as the response of sensor obtained for only cyanide (40.0 µmol L^−1^) was compared with those calculated for a mixture of cyanide and a certain foreign species which are mixing together with the ratio of (1:1). The results are represented in Figure S3. As shown, the presence of foreign species did not have any effects on the determination of cyanide.

**Table 2:** The effect of foreign species on the response of assay to cyanide ions (40.0 µmol L^−1^).

Foreign species	Tolerance^1^ [Foreign species]/[Cyanide]
Na^+^, K^+^, Ca^2+^, Mg^2+^, Fe^3+^, Fe^2+^, Al^3+^, NO_3_^−^, PO_4_^3−^, SO_4_^2−^, CO_3_^2−^, Uric acid, Nicotine, Ethanol, Cholesterol, Cysteine, Glucose	500
Urea, SO_3_^2−^, Br^−^, Cl^−^, ClO^−^	50
I^−^, SCN^−^	20

^1^The tolerance limit is introduce as the amount of foreign species in the present of cyanide leading to a relative error less than 5% in the determination of cyanide.

### Real sample analysis

The ability of the assay in detection and determination of cyanide ions in blood samples was verified. The experiment was performed with the help of normal people, firemen and fire victims. Since cigarette smoking can affect the amount of cyanide, both smokers and non-smokers participated in this study. A sample of 5.0 ml was taken from each participant. Each sample was centrifuged at 5000 g for 7 min to separate the plasma section from the other parts of blood. Additionally, 1.0 µL of the separated plasma was injected into µPAD. The parallel analysis was conducted by GC/MS as a reference method. The results obtained by both analytical techniques were presented in Table 3 and statistically compared to each other. As shown, the values of both relative error and *t* student test were lower than the critical values. It confirmed a good correlation between the results of the developed assay and the reference technique. Therefore, the fabricated µPAD can be a reliable alternative to cyanide measurements in biological samples. As Table 3 shows, the mean of the cyanide concentration in different studied categories were acquired as 2.0, 6.6, 3.4, 9.0, 26.6 and 55.6 µmol L^−1^ for control (non-smoker), control (smoker), firemen (non-smokers), firemen (smokers), non-fatal casualties and fatal casualties, respectively. Based on the mean values, the amount of cyanide in the people who were dead was higher than 20.0 µmol L^−1^. As observed, the cyanide concentration is higher in firemen than in control samples (who were not exposed to fire smoke); however, it is much less than the quantities of cyanide in fire victims. Compared to non-smokers, the level of cyanide was increased in smoker volunteers.

**Table 3:** Determination of cyanide concentration in the blood sample of fire survivors.

Volunteer	Number of sample	Found^1^ (μmol/L)		t_experimental_^3^	Relative error (%)
		Proposed sensor	GC/MS^2^		
Control (Non-Smoker)	1	2.5 (± 0.11)	2.6 (± 0.082)	1.30	− 3.8
	2	1.7 (± 0.063)	1.8 (± 0.050)	2.50	− 5.6
	3	1.3 (± 0.067)	1.2 (± 0.031)	1.82	8.3
	4	2.6 (± 0.12)	2.5 (± 0.088)	2.25	4.0
	5	2.1 (± 0.11)	2.2 (± 0.017)	1.61	− 4.5
Control (Smoker)	1	6.3 (± 0.32)	6.0 (± 0.22)	1.73	5.0
	2	7.1 (± 0.34)	6.7 (± 0.24)	2.15	6.0
	3	5.8 (± 0.30)	6.1 (± 0.26)	1.69	− 4.9
	4	6.5 (± 0.23)	6.3 (± 0.25)	1.32	3.2
	5	7.3 (± 0.30)	7.7 (± 0.34)	1.97	− 5.2
Firemen (non-smokers)	1	3.4 (± 0.16)	3.6 (± 0.12)	2.16	− 5.6
	2	2.9 (± 0.13)	3.1 (± 0.12)	2.53	− 6.4
	3	3.8 (± 0.20)	3.6 (± 0.15)	1.79	5.6
	4	3.6 (± 0.17)	3.4 (± 0.11)	2.21	5.9
	5	3.4 (± 0.18)	3.5 (± 0.15)	0.95	− 2.8
Firemen (smokers)	1	7.8 (± 0.33)	8.2 (± 0.31)	1.98	− 4.9
	2	9.6 (± 0.45)	9.1 (± 0.37)	1.92	5.5
	3	8.5 (± 0.41)	8.0 (± 0.28)	2.25	6.2
	4	10.2 (± 0.49)	9.8 (± 0.33)	1.51	− 4.1
	5	8.7 (± 0.35)	8.3 (± 0.26)	2.05	4.8
Non-fatal casualties	1	22.4 (± 1.01)	21.2 (± 0.67)	2.21	5.7
	2	30.2 (± 1.05)	28.4 (± 1.03)	2.73	6.3
	3	34.0 (± 1.53)	35.7 (± 1.31)	1.88	− 4.8
	4	19.5 (± 1.03)	20.8 (± 0.88)	2.14	− 6.2
	5	27.1 (± 1.28)	28.6 (± 0.82)	2.21	− 5.2
Fatal casualties	1	42.6 (± 2.24)	45.0 (± 1.64)	1.93	− 5.3
	2	69.4 (± 3.28)	65.7 (± 2.15)	2.11	5.6
	3	49.1 (± 1.99)	52.2 (± 1.50)	2.78	− 5.9
	4	55.0 (± 2.84)	51.7 (± 1.63)	2.25	6.4
	5	62.1 (± 2.70)	59.5 (± 1.82)	1.78	4.4

^1^Mean of 5 determination (± SD).

^2^Gas chromatography-Mass spectroscopy.

^3^t_critical (8, 0.05) = 2.31.

The analytical characterizations of the fabricated µPAD were compared to a number of colorimetric sensors to determine cyanide. The published methods used various receptors such as metal complex, bimetallic nanoparticles and organic materials. Thus, the Pt complex has not been used as a colorimetric detector of cyanide ions up to now. As Table S3 shows, the proposed assay shows a good linear range and low-level detection limit compared to the previous methods. Also, the fabricated sensor consumed only 2.0 μL of analyte and detected cyanide in the shortest possible time rather than the other methods.

## Conclusion

This study introduced a simple colorimetric assay using a square-planar platinum (II) complex as a receptor of cyanide ions. The assay was prepared based on the origami art assembling all experimental steps in a piece of paper, used a trace volume of analyte and consumed a few seconds for analysis. These advantages cause µPAD to have better performance than ordinary instrumental methods and other paper-based devices. The receptor showed high affinity to cyanide ions in the presence of other co-existing compounds. The response of sensor has a linear correlation with the cyanide concentrations in the range of 1.0–100.0 µmol L^−1^. The detection limit was calculated as 0.4 µmol L^−1^for this determination. The calibration and analytical sensitivity were equal to 0.8 and 0.3. The proposed µPAD opens a new way to determine the amount of cyanide in water, food and blood samples, being important for both forensic and medical centers to explore the reasons for human death or his suicide.

## Methods

All methods were performed in accordance with the relevant guidelines and regulations.

### Chemicals and materials

All chemical compounds were used in analytical grades without any purifications. Some materials such as potassium nitrate (KNO_3_), potassium cyanide (KCN), sodium nitrate (NaNO_3_), sodium nitrite (NaNO_2_), sodium carbonate (Na_2_CO_3_), sodium sulfate (Na_2_SO_4_), sodium sulfite (Na_2_SO_3_), sodium phosphate (Na_3_PO_4_), sodium thiocyanate (NaSCN), sodium chloride (NaCl), sodium bromide (NaBr), sodium iodide (NaI), sodium hydroxide (NaOH), calcium nitrate tetrahydrate (Ca(NO_3_)_2_.4H_2_O), magnesium nitrate hexahydrate (Mg(NO_3_)_2_ · 6 H_2_O), aluminium nitrate nonahydrate (Al(NO_3_)_3_.9H_2_O), iron (II) sulfate heptahydrate (FeSO_4_.7H_2_O), iron (III) chloride hexahydrate (FeCl_3_.6H_2_O), ethanol, boric acid (H_3_BO_3_), hydrochloric acid (HCl), tris-hydroxymethyl methane (Tris), acetone, n-hexane,1,10-phenanthroline,cysteine, urea, uric acid, cholesterol, glucose, nicotine and trichloroacetic acid (TCA) were purchased from Merck Chemical Company. µPAD was fabricated by Whatman Grade NO.2 filter paper. The stock solution of cyanide ions was prepared with the concentration of 150.0 µmol L^−1^. Tris and borate buffer were made at the concentration of 0.2 M, and their pH was adjusted at an optimum value using NaOH (1.0 M) and HCl (1.0 M).

### Apparatus and software

UV/Vis spectrophotometer (JASCO, Model V-570) and field emission scanning electron microscopy (FE-SEM; MIRA3 TESCAN) were used to characterize the paper device. Brand type micropipettes were employed for injection of samples. The pH of media was adjusted by a Metrohm 632 pH-meter (Model 780 pH lab). The schematic pattern of µPAD was drawn by the AutoCAD 2016 software (https://www.autodesk.com/products/autocad). A HP Laser Jet printer 1320 w was performed for printing pattern utilized to print patterns on the paper. The printer was equipped by a HP Q5949A toner cartridge with a resolution of 600 dpi. An oven (MEMMERT UN 30) was used to bake the paper. The sensor photographs were recorded by a smartphone (Samsung Galaxy A7). The image analysis was conducted by the Image J software (1.51n, National Institutes of Health, USA) (https://imagej.nih.gov/ij/download.html) and MATLAB R2015 scientific software (https://www.mathworks.com).

### Synthesis of receptor

[Pt (*p*-MeC_6_H_4_)_2_(phen)] (Compound A) was used as a colorimetric sensing element in this study. The synthesis procedure and characterization of recognition element are described in the supporting information document (Sect. 2).

### µPAD preparation

Figure 1a shows the pattern designed by the AutoCAD software. It is a rectangle with a dimension of 1 × 5 cm and made of two parts, including circle shaped hydrophilic zones (white sections) and a hydrophobic barrier (black section). The diameter of each circle was equal to 0.7 cm. Five zones are embedded in this pattern, which are distinct from each other using a line. Each zone is drawn for a specified task consisting of sample injection, sample acidification, gas collection, gas alkalization and analyte detection. The pattern is printed on a Whatman filter paper and then baked in the oven at 180 °C for 45 min^37^. Thorough this process, the printer ink is immobilized on the paper surface, and then melt and penetrated in the paper texture^38^. This work is performed to create a barrier with high hydrophobicity around the hydrophilic zones. Figure 1b presents the image of the fabricated µPAD.

### Colorimetric determination

Each hydrophilic zone was filled by respective reagent: acidification zone was poured by TCA (25.0% W/V), borate buffer (0.1 M, pH 10.0) was added to the alkalization zone and finally, the Pt complex as a receptor was injected into the detection zone. Each zone contained 1.0 µL of reagent. Furthermore, the gas collection zone was empty at this time. The µPAD was folded from the marked lines such that injection and detection zones were the top and bottom layers of the device, respectively. A paper holder was used to make the layers fit perfectly together. This holder is two layers of wood fastened together by a peg. There is a hole in the top layer for injecting the samples. Moreover, 2.0 µL of the cyanide containing sample was subjected to µPAD from the injection zone. The sample flowed through the layers of µPAD and reached the detection zone. It interacted with the receptor and changed the color of the receptor from yellow to being colorless. This interaction can be influenced by TCA concentration, pH of media, type of buffer and its concentration, amount of receptor, volume of sample and time required for interaction. Therefore, these parameters need to be optimized.

### Image analysis

**Scheme 1:**
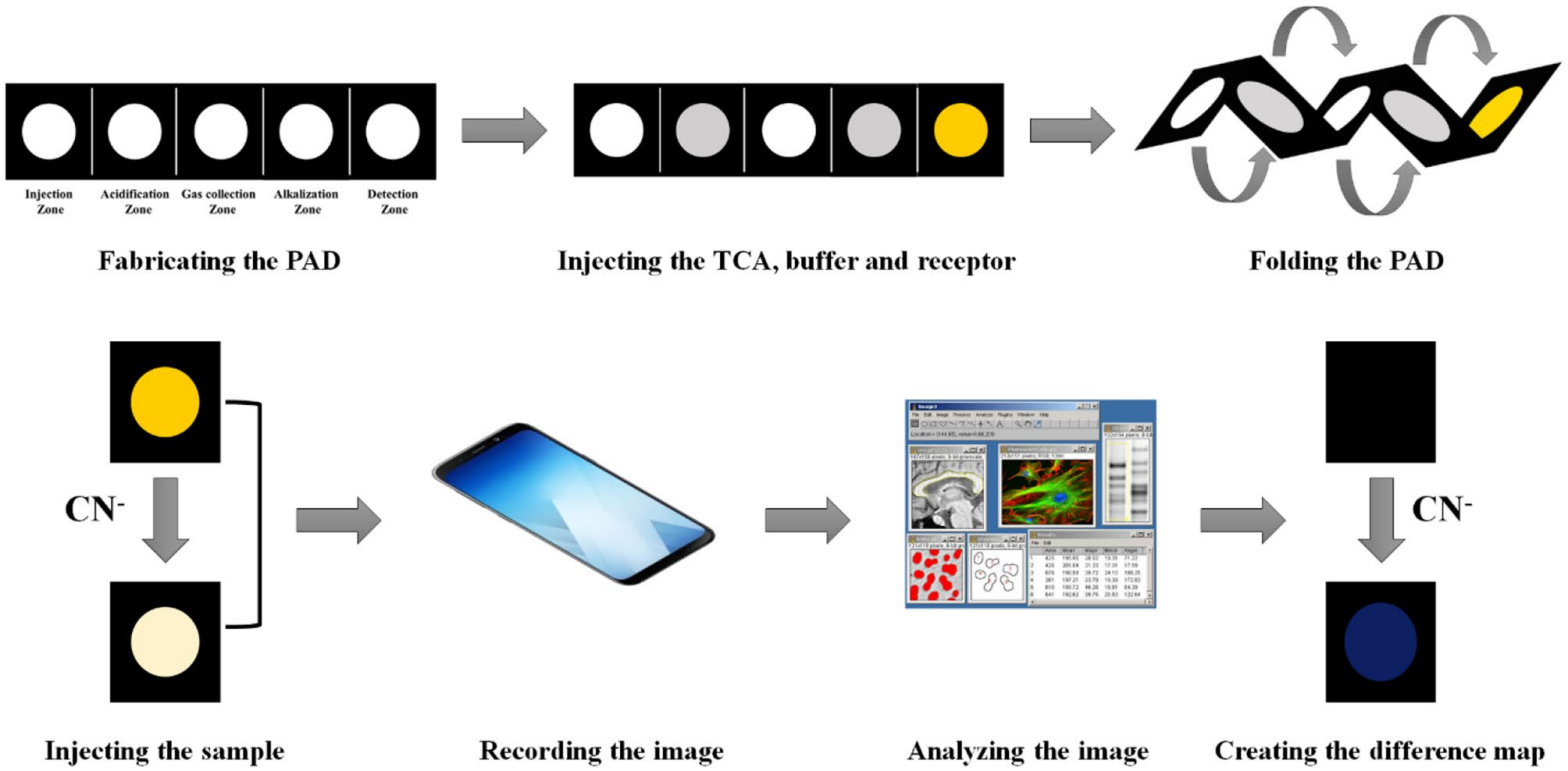
Summary of the experimental procedure for detection of cyanide ions.

Changes in the color of the detection zone were monitored by a smartphone. To remove the effect of ambient light, the experiment was performed in a laboratory-made box containing two lamps (8 W). This box is a wooden cube with dimensions of 30 cm. The lamps are placed on the roof of this box such that they have a 45 degree angle with the location of the sample. The image of the sensor before and after exposure to analyte was recoded and saved with the name of “reference” and “response” images. Each image was uploaded to the Image J software. The whole area of the detection zone was selected. The software determined the intensities of R and G and B for pixel by pixel and finally the average of these intensities were calculated for each color elements. The difference between the color values of reference and response images was determined. For each analysis, three color values related to red, green and blue color elements were collected in a data vector. The Euclidean norm of the response vector was calculated as the pure response for further study:

$$Euclidean \,norm= \sqrt{{(\Delta R)}^{2}+{(\Delta G)}^{2}+{(\Delta B)}^{2}}$$

where $\Delta R$, $\Delta G$ and $\Delta B$ are the difference color values for red, green and blue elements. Scheme 1 depicts the schematic diagram for the general process of detection.

The data vector containing the numerical color elements of R, G and B was converted to color circle shape with the help of image processing method in the MATLAB software.

### Determination of blood cyanide concentration

#### Use of human participants

##### Identifying information

The participants were divided into six groups, including healthy people as control samples (smokers and non-smokers), firemen (smokers and non-smokers), non-fatal casualties and fatal casualties. The individuals were asked to read the proposal and filled the consent form. The informed consent was obtained. This form contains the definition of study, its cost, advantages, limitations and sampling procedures. In this form, we stated the personal information and medical documents for each participant would be kept confidential. Also, each person could refuse to participate in this study or leave the test at any time. The proposal for this study was approved by the Medical Ethics Committee of Ahvaz University of Medical Sciences (Ethics code: IR.AJUMS.REC.1398.337). In this proposal, all methods including fabrication of sensor, detection procedure, statistical and confirmation analysis was completely explained.

##### Experimental procedure

The µPAD was applied to determine the amount of cyanide ions in blood sample. The volunteers were selected from fire victims and healthy peoples. Furthermore, the effect of smoking on BCC was investigated. They were given us their blood samples collected in a sterile tubes. To prevent the blood clotting, it was mixed with heparin. The plasma portion of blood was separated and used for further studies. The plasma samples were partitioned in two identical parts: half of sample was analyzed by GC/MS, and the rest was examined by the fabricated µPAD. In our study, the experiment was performed as it was conducted in the previous section. The results obtained by the colorimetric method were compared to those achieved by the standard assay with the help of statistical parameters.

## Supplementary Information

Supplementary Information.
