# Supplementary material for: Origami paper analytical assay based on metal complex sensor for rapid determination of blood cyanide concentration in fire survivors
Source: Sci Rep. 2021 Feb 10;11:3521. doi: 10.1038/s41598-021-83186-0 (PMC7876125; doi:10.1038/s41598-021-83186-0)
Supplement: Supplementary file 1 — Supplementary Information. [file 41598_2021_83186_MOESM1_ESM.docx]

Supporting Information

for

**Origami paper analytical assay based on metal complex sensor for rapid determination of blood cyanide concentration in fire survivors**

Azarmidokht Sheini^a,*^, Marzieh Dadkhah Aseman^b^, Mohammad Mahdi Bordbar^c^

^a^ Department of Mechanical Engineering, Shohadaye Hoveizeh University of Technology, Susangerd, 78986, Iran.

^b^ Department of Chemistry, Faculty of Sciences, Tarbiat Modares University, Tehran, 4838, Iran..

^c^ Independent researcher, Personal laboratory, Fasa, 74614, Iran.

Email address:

[noyanchem2019@gmail.com](mailto:noyanchem2019@gmail.com)

[azar.sheini@gmail.com](mailto:azar.sheini@gmail.com)

**1. Synthesis of precursor**

The precursor *cis*-[Pt(*p*-MeC_6_H_4_)_2_(SMe_2_)_2_], was prepared by the literature methods ^1^. In a typical experiment, a freshly prepared solution of para-tolyllithium (4 ml), was added slowly to a stirred, ice-cooled solution of finely powdered *cis*/*trans*-[PtCl_2_(SMe_2_)_2_] (1 g; 2.6 mmol) in dry ether (30 ml). The reaction mixture was stirred for 2 h at 0 °C and subsequently hydrolyzed with a few ml iced water. Separation of the organic layer, extraction with dichloromethane, drying over MgSO_4_ and evaporation gave *cis*-[Pt(*p*-MeC_6_H_4_)_2_(SMe_2_)_2_]. The solvent was evaporated and the residue was washed with a few ml of cooled acetone, the product is obtained as a white solid. Yield: 0.95 g; 73 %, mp = 147-151 °C (decomp.). NMR data in CDCl_3_: δ (^1^H) = 2.11 (s, ^3^*J*(PtH) = 23.9 Hz, 12H, SMe_2_ ligands); 2.14 (s, 6H, Me groups of the *para-*tolyl ligands); 6.75 (d, ^3^*J*(HH) = 7.9 Hz, 4H, H_m_ of *para*-tolyl ligand); 7.23 (d, ^3^*J*(PtH) = 71.8 Hz, ^3^*J*(HH) = 8.0 Hz, 4H, H_o_ of *para*-tolyl ligand).

**2. Synthesis of receptor**

[Pt (*p*-MeC_6_H_4_)_2_(phen)] (Compound **A**) was used as a colorimetric sensing element in this study. This complex was synthesized according to the reported procedure ^2,3^. Briefly, [Pt(p-MeC_6_H_4_)_2_(SMe_2_)_2_] (504 mg, 1 mmol) was dissolved in diethyl ether (20 mL), and 1,10-phenanthroline (180mg,1mmol) was added to it. The mixture was stirred at room temperature for 2 h, after which the solution turned yellow. The solvent was evaporated, and the yellow residue was washed with n-hexane and dried under vacuum. Their characterizations were performed by the elemental analysis and nuclear magnetic resonance (NMR) spectroscopy, which were represented in previous researches ^2,3^ and are summarized as follows: Yield: 95%; mp 288 °C (dec). Anal. Found: C, 55.57; H, 3.91; N, 4.85. C_26_H_22_N_2_Pt requires: C, 56.01; H, 3.98; N, 5.02. ^1^H NMR:δ 2.30 (6H, s, ArCH_3_), 6.95 (4H, d, ^3^*J*(H_o_H_m_) = 7.8 Hz, H_m_ of Ar), 7.48 (d, 4H, ^3^*J*(H_m_H_o_) = 7.8 Hz, ^3^*J*(PtH_o_) = 68.7 Hz, H_o_ of Ar), 9.02 (d, 2H, ^3^*J*(H_2_H_3_) = 5.0 Hz, ^3^*J*(PtH_2_) = 18.6 Hz, H_2_ and H_9_ of phen), 8.55 (d, 2H, ^3^*J*(H_3_H_4_) = 8.2 Hz, H_4_ and H_7_ of phen), 7.97 (s, 2H, H_5_ and H_6_ of phen), 7.77 (dd, 2H, ^3^*J*(H_4_H_3_) = 8.2 Hz, ^3^*J*(H_2_H_3_) = 5.0 Hz, H_3_ and H_8_ of phen). The element analysis of this compounds was investigated by energy-dispersive X-ray spectroscopy. The result states that the amount of Platinum in this compounds is equal to 30 %.

Figure S1a presents the chemical structure of the synthesized receptor. For further studies, the appropriate amount of this material was dissolved in the specified volume of acetone.

**3. Analysis condition for GC/MS**

Determination of cyanide concentration in blood samples was performed by the GC/MS method with help of the following conditions: GC column: BR-5ms (30 m × 0.25 mm, 0.25 µm, BRUKER, USA), carrier gas: helium, flow rate: 1.0 mL.min^-1^, injector temperature: 210°C, injection volume: 1.0 µL, split Ratio: 1:5, ionization: Electron ionization (EI), source temperature: 200°C, transfer line temperature: 250°C, retention time (min): 6.78 ^4^.

**4. Calculation of detection limit**

In this study, the theoretical detection limit was calculated by following procedure. First, the calibration equation was obtained. Then, the concentrations of cyanide was subjected to this equation. Therefore, a theoretical response was obtained for each concentration. The difference between theoretical ($\hat{y})$ and experimental ($y)$ responses was calculated for each concentration. The result was used to determine the standard deviations of regression with the help of following equation:

$$S_{\frac{y}{x}}=\sqrt{\frac{\sum_{i} {(y_{i}-\hat{y}_{i})}^{2}}{n-2}}$$

In this equation, *i* indicates a specified concentration and n is a total number of concentration that used in this study. This statistical parameter was used for calculating the limit of detection which is obtained by following equation:

$$Detection limit=\frac{3 S_{\frac{y}{x}}}{m}$$

The parameter (m) in the above equation is the slope of calibration plot.

**References**

1. Rashidi, M., Hashemi, M., Khorasani-Motlagh, M. & Puddephatt, R. J. Aryldiplatinum(II) complexes containing dimethyl sulfide and bis(diphenylphosphino)methane as bridging ligands. *Organometallics* **19,** 2751–2755 (2000).

2. Rashidi, M., Nabavizadeh, M., Hakimelahi, R. & Jamali, S. Kinetics and mechanism of cleavage of the oxygen-oxygen bond in hydrogen peroxide and dibenzoyl peroxide by arylplatinum(II) complexes. *J. Chem. Soc. Dalt. Trans.* 3430–3434 (2001). doi:10.1039/b103690b

3. Habibzadeh, S. *et al.* Steric and solvent effects on the secondary kinetic α-deuterium isotope effects in the reaction of methyl iodide with organoplatinum(ii) complexes: Application of a second-order technique in measuring the rates of rapid processes. *Organometallics* **29,** 82–88 (2010).

4. Shin, M. C., Kwon, Y. S., Kim, J. H., Hwang, K. & Seo, J. S. Validation of an analytical method for cyanide determination in blood, urine, lung, and skin tissues of rats using gas chromatography mass spectrometry (GC-MS). *Anal. Sci. Technol.* **32,** 88–95 (2019).

5. Bortey-Sam, N. *et al.* Diagnosis of cyanide poisoning using an automated, field-portable sensor for rapid analysis of blood cyanide concentrations. *Anal. Chim. Acta* **1098,** 125–132 (2020).

6. Khajehsharifi, H. & Bordbar, M. M. A highly selective chemosensor for detection and determination of cyanide by using an indicator displacement assay and PC-ANN and its logic gate behavior. *Sensors Actuators, B Chem.* **209,** 1015–1022 (2015).

7. Männel-Croisé, C. & Zelder, F. Rapid visual detection of blood cyanide. *Anal. Methods* **4,** 2632–2634 (2012).

8. Chaudhary, M. T. *et al.* Rapid and Economical Colorimetric Detection of Cyanide in Blood Using Vitamin B12. *Aust. J. Forensic Sci.* **48,** 42–49 (2016).

9. Hu, G., Yang, C., Liu, H. & Shen, J. Pillar[5]arene-functionalized paper as a fluorescent sensor for cyanide ions in water. *New J. Chem.* **43,** 11473–11476 (2019).

10. Khatha, P. *et al.* Distance-based paper device combined with headspace extraction for determination of cyanide. *Sensors (Switzerland)* **19,** (2019).

11. Erdemir, S. & Malkondu, S. On-site and low-cost detection of cyanide by simple colorimetric and fluorogenic sensors: Smartphone and test strip applications. *Talanta* **207,** (2020).

(a)

(b)

**Figure S1.** (a) The chemical structure of receptor before (A) and after (B) interaction with cyanide ions, (b) The chemical reaction equation for cyanide detection.


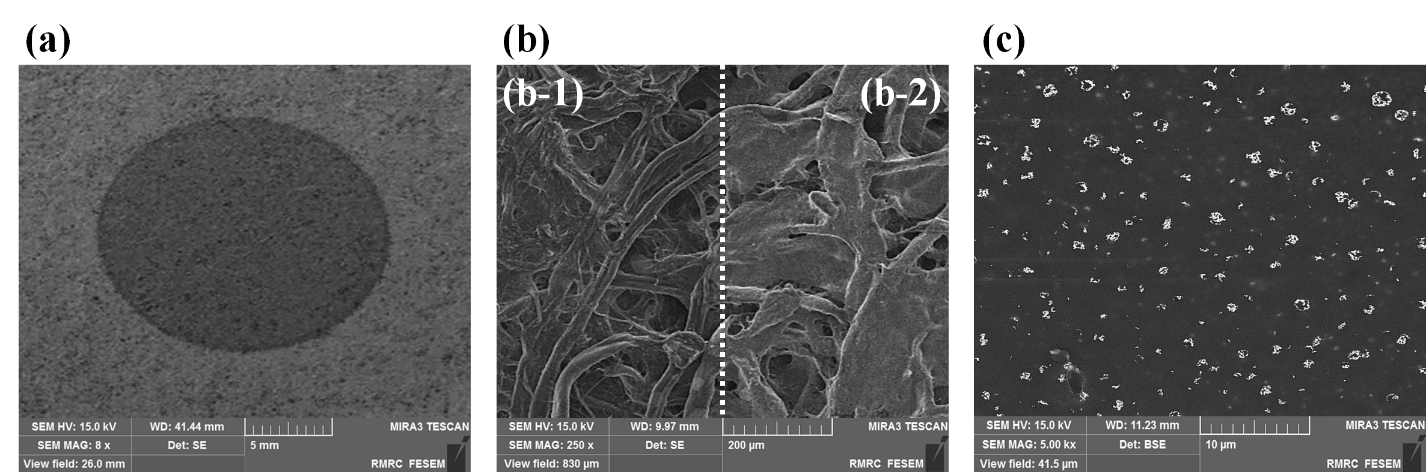


**
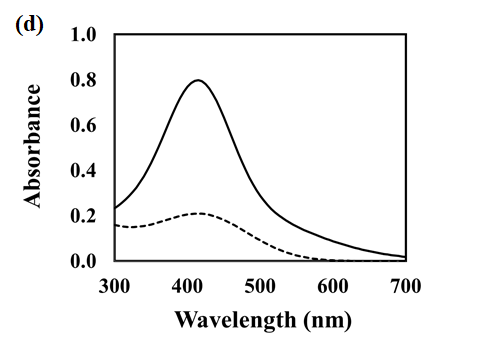
**

**Figure S2.** The SEM image of detection zone. (a) The whole area of detection zone which is coated by 1.0 µL of receptor, (b-1) the structure of hydrophilic zone constructed by interwoven cellulose fibers, (b-2) the structure of hydrophobic barrier indicated that the pores of paper was covered and blocked by ink printer, (c) the homogenous distribution of receptor on the paper. (d) The absorbance spectra of receptor before (solid line) and after (dashed line) interaction with cyanide ions (100.0 µmol.L^-1^). The study was performed in the presence of borate buffer (0.1 M, pH 10.0).


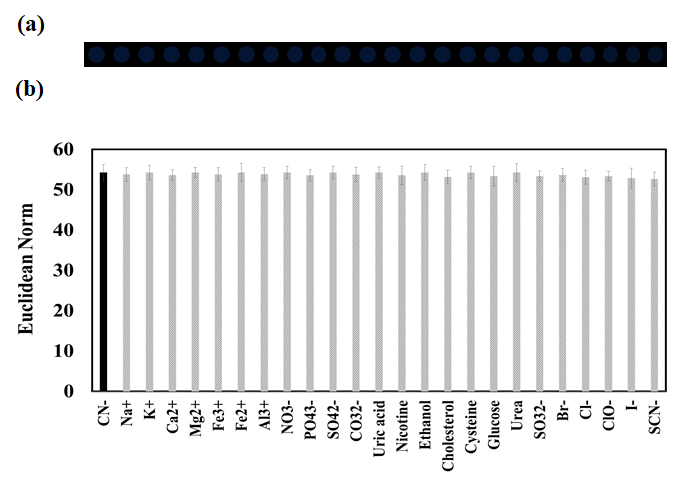


**Figure S3.** The effect of the foreign species on the response of sensor in the presence of cyanide. The response of sensor are shown by (a) colorimetric sensor array and (b) numerical plot. The numerical plot indicates the Euclidean norm of sensor for cyanide (black bar) and the mixture of cyanide and a certain foreign specie (gray bar). The concentration of both cyanide and the foreign species were equal to 40.0 µmol.L^-1^.

**Table S1.** Evaluation of the repeatability for detection zone fabrication

| **Color element** | **Sensor 1** | **Sensor 2** | **Sensor 3** | **Sensor 4** | **Sensor 5** | **RSD %** |
| --- | --- | --- | --- | --- | --- | --- |
| **Red** | 213 | 214 | 214 | 213 | 212 | 0.39 |
| **Green** | 176 | 177 | 177 | 176 | 174 | 0.70 |
| **Blue** | 101 | 104 | 104 | 101 | 98 | 2.47 |

**Table S2.** Comparison between the response of sensor immediately and 20 days after PAD fabrication

| **Euclidean norm** | | **t_experimental_^2^** | **Relative error (%)** |
| --- | --- | --- | --- |
| **After sensor fabrication** | **20 days after making the sensor** |  |  |
| 54 (±2.04) | 52 (±1.8) | 1.57 | -3.7 |
| ^1^Mean of 5 measurement (±SD)  ^2^ t_critical (8,0.05) = 2.31 | |  |  |

**Table S3.** Comparison between the characterizations of optical sensors for detection of cyanide

| **Media** | **Receptor** | **Linear range (µmol.L^-1^)** | **Detection limit**  **(µmol.L^-1^)** | **Real sample** | **Volume of analyte (μL)** | **Response time (Second)** | **Interference** | **Ref.** |
| --- | --- | --- | --- | --- | --- | --- | --- | --- |
| Solution | Naphthalene dialdehyde and taurine | 10.0-200.0 | 5.0 | Blood | 25.0 | 60 | None | ^5^ |
| Solution | Zn complex | 6.6-265.0 | 0.2 | Blood | - | 15 | None | ^6^ |
| Paper | Co complex | 0.0-50.0 | 3.0 | Blood | 500.0 | 900 | None | ^7^ |
| Paper | Vitamin B12 | 38.4-961.0 | 38.4 | Blood | 2000.0 | 300 | None | ^8^ |
| Paper | Pillar[5]arene derivative | 0.1-1000.0 | 0.1 | - | - | 30 | None | ^9^ |
| Paper | Au@Ag BimetallicNPs | 1.9-38.4 | 0.38 | water | 50.0 | 2400 | None | ^10^ |
| Paper | Organic compound | 5.0-35.0 | 3.2 | Food | - | - | None | ^11^ |
| Paper | Pt complex | 1.0-100.0 | 0.43 | Blood | 2.0 | 25 | None | This work |
